# Extraskeletal Ewing’s sarcoma/primitive neuroectodermal tumor of the mediastinum: Significant response to chemoradiotherapy

**DOI:** 10.3892/ol.2014.2788

**Published:** 2014-12-10

**Authors:** MIN LIU, BAILONG LIU, LIHUA DONG, TAO HAN, LEI ZHANG

**Affiliations:** 1 Department of Radiation Oncology, The First Hospital, Jilin University, Changchun, Jilin 130021, P.R. China; 2 Department of Radiology, The First Hospital, Jilin University, Changchun, Jilin 130021, P.R. China

**Keywords:** extraskeletal Ewing’s sarcoma, mediastinum, chemoradiotherapy

## Abstract

Primary mediastinal extraskeletal Ewing’s sarcoma (EES) is quite rare. To the best of our knowledge, only five cases have been reported. Given the paucity of data, there is consequently no optimal treatment strategy available. The current study presents the case of a 51-year-old female with unresectable EES of the posterior mediastinum. Chemoradiotherapy achieved near complete remission without severe side-effects. The literature associated with EES is also reviewed. The present case highlights the possibility of the diagnosis of EES for a mediastinal mass. Chemoradiotherapy may be a good option for unresectable cases. In the future, large-scale collaborative clinical trials should be initiated to provide an improved understanding of the characteristics of EES and the best treatment strategy.

## Introduction

Extraskeletal Ewing’s sarcoma (EES)/primitive neuroectodermal tumor (PNET) is a rare entity, accounting for 15% of all Ewing’s sarcomas (1). The most common clinical manifestation of this disease is a rapidly growing, painful lump (2). Pathological, cytogenetic, immunohistochemical and molecular genetic analysis contribute to an accurate diagnosis (3). Multidisciplinary treatment modalities comprising extended resection, aggressive chemotherapy and local irradiation are recommended (4). At present, EES/PNET is viewed as a potentially curable disease (5).

Primary mediastinal EES is extremely rare. The present study describes the case of a middle-aged female with mediastinal EES who received sequential chemotherapy and radiotherapy and achieved a marked response. Written informed consent was obtained from the patient. Additionally, the associated studies on EES are also reviewed. Further studies are required to establish the standard treatment strategy for EES. Written informed consent was obtained from the patient.

## Case report

On December 25, 2012, a 51-year-old female presented with intermittent chest pain that had been apparent for one year. The physical examination was unremarkable. The patient’s performance status was 1 according to an Eastern Cooperative Oncology Group (ECOG) evaluation (6).

**Figure 1:**
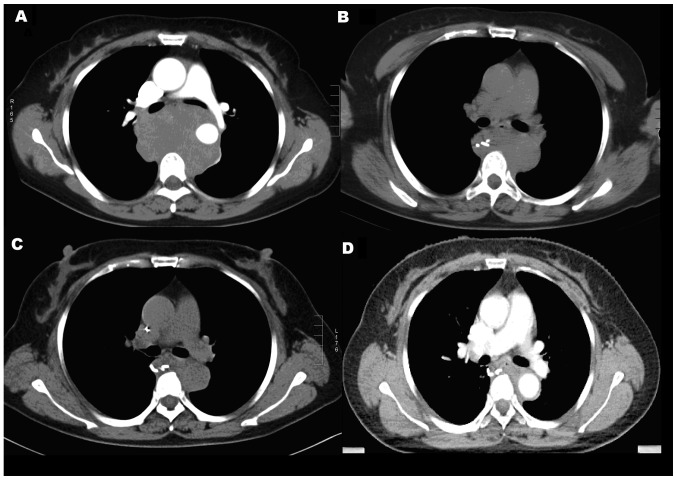
Thoracic computed tomgraphy (CT) scan showing the lesion prior to treatment and during varying stages of treatment. (A) CT scan from December 25, 2012, showing a large mass in the posterior mediastinum. (B) Following one cycle of chemotherapy, the tumor was greatly reduced in size. (C) CT scan showing the smaller tumor following three cycles of chemotherapy. (D) Pre-radiotherapy CT scan showing the residual tumor.

**Figure 2:**
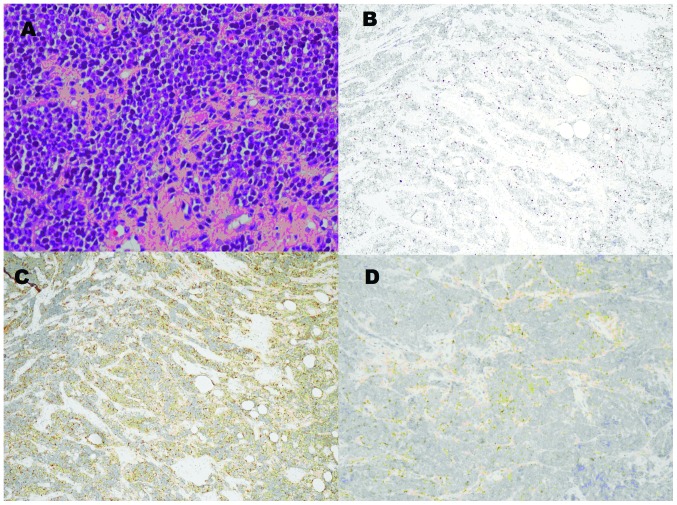
Pathological and immunohistochemical results. (A) The lesion was composed of primitive small round cells (H&E staining), with (B) a Ki-67 of 20% and positivity for (C) cluster of differentiation 99 and (D) synaptophysin.

Laboratory investigations revealed a normal complete blood cell count, coagulation routine and serum biochemical profile. The thoracic computed tomography (CT) scan revealed a huge mass in the posterior mediastinum, with invasion of the esophagus, descending aorta and right pulmonary artery (Fig. 1A). On December 31, 2012, a biopsy of the mediastinal mass was performed by thoracoscopy, during which an irregular posterior mediastinal mass measuring ~8×8 cm was located under the arch of the azygos vein. The final pathology showed a malignancy of small round cells, which was consistent with an Ewing’s sarcoma/PNET (Fig. 2A). The immunohistochemical results demonstrated a Ki-67 of 20% (Fig. 2B), positivity for cluster of differentiation (CD)99 (Fig. 2C) and synaptophysin (Fig. 2D), partial positivity for CD56, neuron-specific enolase and S-100, and negativity for CD1a, Wilms tumor 1 protein, octamer-binding transcription factor 3/4, vimentin, epithelial membrane antigen, cytokeratin (CK)5/6, desmin, thyroid transcription factor-1, inhibin A, CD34, terminal deoxynucleotidyl transferase, CK and leukocyte common antigen. The following abdominal CT, brain magnetic resonance imaging and bone scan excluded the possibility of metastasis.

The patient was administered four cycles of dacarbazine and pirarubicin chemotherapy. One cycle consisted of 300 mg dacarbazine on days 1–5 and 40 mg pirarubicin on days 1–2, for 21 days (patient’s body surface area, ~1.6 m^2^). Following one cycle of chemotherapy, the tumor rapidly decreased in size (Fig. 1B) and the patient’s discomfort disappeared. The thoracic CT revealed that the tumor became even smaller after three cycles compared with one cycle (Fig. 1C). Minimal residual tumor remained subsequent to four cycles of chemotherapy (Fig. 1D). Radiotherapy was then administered to the tumor bed at a total dose of 54 Gy/30 fractions over 42 days (between May 10, 2013, and June 21, 2013) and the recorded side-effects, such as esophagitis and leukopenia, were mild.

## Discussion

EES is a rare entity with high-grade malignancy commonly involving the soft tissues of the trunk and extremities. The thoracic sites of EES include the chest wall, trachea, spinal epidural space, paraspinal area and mediastinum (7–11). Primary mediastinal EES/PNET is extremely rare (1,12); to the best of our knowledge, only five such cases have previously been reported (1,11–14). The present case will therefore aid in expanding our understanding of this distinct neoplasm.

EES often exhibits translocation of (11;22)(q24;q12) (3). CD99-positive expression plays a crucial role in the diagnosis of EES (15). Tural *et al* reported indicators of poor overall survival, namely a primary tumor of >8 cm, a high level of lactic dehydrogenase, metastasis at the time of the first hospital visit, a poor response to chemotherapy, radiotherapy as the single method to improve local control and positive margins (16).

For a long period of time, EES had been regarded as exhibiting no significant differences to osseous Ewing’s sarcoma. A study by Applebaum *et al* (17) was the first to reveal that EES exhibited a different therapeutic response and clinical characteristics. The mean age of onset is older in EES, with a biphasic distribution to its peak age of onset: >35 and <5 years. Furthermore, the preponderance of males is less marked in EES compared with Ewing’s sarcoma of the bone. Axial locations are more likely than the pelvic cavity. Additionally, more patients with EES receive radiotherapy.

EES is highly aggressive. Local relapse and distant metastases are frequent. Multidisciplinary measures are important for improvements in survival. A retrospective study of 24 cases of EES showed a 61% five-year overall survival rate following multimodality therapies (18). In a study by Lee *et al* (7), two cases of EES of the chest achieved at least 30 and 22 months progression free survival, respectively, following comprehensive treatment. The two cases utilized extended resections and a post-operative alternate chemotherapy regimen of vincristine, Adriamycin and cyclophosphamide, and ifosfamide and etoposide (IE). One patient also received 54 Gy/30 fractions of radiotherapy to the tumor bed (7). Extended resection combined with multidrug chemotherapy often results in a clinical benefit (11). However, from a review of the literature, it can be observed that no consensus has yet been reached with regard to a standard chemotherapy regimen. Tural *et al* recommended an intense strategy of administering vincristine, Adriamycin, cyclophosphamide and actinomycin D alternately with IE (16). Tao *et al* held the view that the use of platinum-based chemotherapy should be considered (4). In the present case, dacarbazine and pirarubicin achieved a good response and the side-effects were mild.

In terms of local control, no random trials have yet been initiated to demonstrate whether surgery combined with radiotherapy is superior to surgery alone. However, complete resection plus radiotherapy is known to have an advantage over radiotherapy. For unresectable cases or tumors that cannot be removed completely, radiotherapy with a radical dose of 50–60 Gy should be performed (1).

In conclusion, the present study describes a relatively rare case of primary mediastinal EES/PNET that could not be resected. Sequential chemotherapy and radiotherapy achieved a favorable response with mild side effects. We propose that dacarbazine and pirarubicin chemotherapy should be considered for unresectable EES/PNET cases for its beneficial effect, and chemoradiotherapy is a good treatment option for such cases. Successful published protocols using collaborative clinical trials will aid in the development of a standard treatment strategy.
